# Further Insights Into the Beck Hopelessness Scale (BHS): Unidimensionality Among Psychiatric Inpatients

**DOI:** 10.3389/fpsyt.2020.00727

**Published:** 2020-07-31

**Authors:** Michela Balsamo, Leonardo Carlucci, Marco Innamorati, David Lester, Maurizio Pompili

**Affiliations:** ^1^Department of Psychology, Health and Territory, School of Medicine and Health Sciences, “G. d’Annunzio” University of Chieti-Pescara, Chieti, Italy; ^2^Dipartimento di Scienze Umane, Università Europea di Roma, Rome, Italy; ^3^Stockton University, Galloway, NJ, United States; ^4^Department of Neurosciences, Mental Health and Sensory Organs, Suicide Prevention Center, Sant’Andrea Hospital, Sapienza University of Rome, Rome, Italy

**Keywords:** hopelessness, inpatients, depression, Mokken analysis, unidimensionality

## Abstract

Short versions of the Beck Hopelessness Scale have all been created according the Classical Test Theory, but the use and the application of this theory has been repeatedly criticized. In the current study, the Item Response Theory approach was employed to refine and shorten the BHS in order to build a reasonably coherent unidimensional scale whose items/symptoms can be treated as ordinal indicators of the theoretical concept of hopelessness, scaled along a single continuum. In a sample of 492 psychiatrically hospitalized, adult patients (51.2% females), predominantly with a diagnosis of Bipolar Disorder type II, the BHS was submitted to Mokken Scale Analysis. A final set of the nine best-fitting items satisfied the assumptions of local independency, monotonicity, and invariance of the item ordering. Using the ROC curve method, the IRT-based 9-item BHS showed good discriminant validity in categorizing psychiatric inpatients with high/medium suicidal risk and patients with and without suicide attempts. With high sensitivity (>.90), this newly developed scale could be used as a valid screening tool for suicidal risk assessment in psychiatric inpatients.

## Introduction

Hopelessness is an important psychological construct, defined as negative expectations regarding oneself and one’s future life and a negative emotional state characterized by the lack of finding a solution for one’s problems (1). In his research focused on depression mood and suicidal behavior, Beck (2–5) observed that patients diagnosed with depressive disorders shared common cognitive features—a negative view of the self, and of the self in relation to the world and in relation to the future. He paid special attention to one of these cognitive features—a negative view of the self in relation to the future, by introducing the construct of “hopelessness”. Unlike depression, it is oriented towards the future as opposed to the present state (6).

According to Beck and his associates, hopelessness has substantial clinical utility for suicide risk assessment and prediction of future suicidal behavior. They produced empirical evidence for the association between hopelessness and suicidality by arguing that severity of suicidal intent is more strongly related to hopelessness compared to depression (5, 7–11).

Subsequently, research showed that hopelessness could lead to suicidality (12–14). As a modifiable key psychological risk factor in suicidal behavior, with an impact that can be reduced by means of appropriate psychotherapeutic interventions, the recognition and assessment of hopelessness plays an important role in the prevention of suicidal behavior (15–17).

### Measurement of Hopelessness: The Beck Hopelessness Scale

To investigate better the construct of the hopelessness, Beck (4), Beck, Weissman, et al. (6) constructed the Beck Hopelessness Scale (BHS). In its development, Beck grouped 9 items from an unpublished inventory assessing the attitudes about the future and 11 items drawn from a set of pessimistic statements formulated by patients with psychiatric diagnoses (6). The BHS scores were found to be strongly correlated with clinical ratings of hopelessness by Beck and colleagues in their validation study (6).

Several studies have indicated good predictive validity for the BHS (11, 14, 18–20). For instance, the BHS was found to predict suicidal thoughts and attempts among 289 psychiatrically hospitalized suicidal youth across a 1–6-month follow-up after hospital discharge (21, 22). Hopelessness, as measured by the BHS, was found a significant predictor of attempted suicides among psychotic patients at first admission to hospital (18).

The BHS performed similarly across inpatients and outpatients, for both psychiatric and medical samples (23–25), and can also be used for predicting social functioning and general status health in psychiatric samples (26).

### Overview on Dimensionality BHS

In order to determine the dimensionality of the scale, Beck, Weissman, et al. (6) subjected the items of the BHS to a factor analysis. The factors were labeled “Feelings about the Future”, “Loss of Motivation”, and “Future Expectations”, respectively. According to the authors, although the factor structure of the BHS made sense clinically, it can vary according to the type of clinical sample being studied and the type of factor-analytic methods conducted. Further studies analyzed this factor structure across different samples (27–30). According to the review by Aish et al. (31), factor structures found in the literature could be grouped as follows: (1) one-factor models (32); (2) two-factor models (33–38); (3) three factor models (27, 29, 30); and (4) models with four or more factors (28). In the reported studies, the emerging factors found differed from those identified by Beck et al.’s study in terms of the assigned factors’ labels and their item composition.

In addition, some authors noted that some original items could fit models different from those proposed (31, 32, 36). For example, Aish and Wasserman (31) found that no strong evidence supported the multidimensionality of the BHS by the first CFA. In detail, 15 items tapped a single dimension of hopelessness, and so a reduced number of 4 items could summarize most of the information contained by the BHS. Thus, the dimensionality of the BHS remains an open issue of considerable interest. For example, Hill, Gallagher, et al. (27) found that only one component(giving up - the motivational component) was significantly related to suicidal intent. In this case, combining different dimensions into a composite scale (39) might reduce the predictive validity of the BHS.

### Short Versions of the BHS

The length of the BHS could be discouraging for the respondents. Lengthy questionnaires reduce data quality and respondent willingness (21), especially in clinical populations (40, 41). In order to be useful in practical settings, an instrument should be sufficiently brief and easy to complete (42, 43), especially when multiple measurement scales are employed.

Previous methodological studies have suggested that a reduction by about 50–70% of the number of items could not compromise substantially the original psychometric functioning of a scale (44–47). This is also true for the BHS (31, 48). For example, some researchers have suggested that a single item, “My future seems dark to me” (item #7), could be sufficient to assess hopelessness. According to Aish and Wasserman (31), this sentence is ideal for summarizing the construct under investigation: the perception of a menacingly ambiguous and hopeless future. This suggestion was supported by Perczel Forintos et al.’s (13) study whose results showed that this item had the highest item-residue correlation (*r* = .75), that is the highest correlation with the total score of the BHS.

Other researchers have investigated the psychometric properties of different 3- or 4-item versions of the BHS. Based on confirmatory factor analysis, Aish and Wasserman (31) reported excellent fit for a 4-item version of the BHS(composed of items #6, 7, 9, 15). In a cross-sectional survey, Yip and Cheung (1) administered this shortened version to some 2,000 Chinese subjects. A significantly high correlation (*r* = .88, *p* <.001) of the shortened version with the original 20-item BHS was found, suggesting that the abbreviated scale can be reliably used in clinical studies. They also reported that the 4-item BHS was able to differentiate patients with and without suicidality, similarly to the original version of the BHS. Recently, Aloba, Akinsulore (48) introduced a new 4-item version of the BHS (composed of items 8, 9, 13, and 15) in a sample of 327 Nigerian adult psychiatric outpatients. The authors reported satisfactory reliability and validity, comparable to that of the long form of the BHS. Other researchers (28, 37) have also suggested that a three-item version of the BHS (items 7, 14, and 20)could represent the scale and be a valid measure of hopelessness.

Finally, some researchers have devised brief modified versions of the BHS. For example, Perczel Forintos, Sallai (13) proposed a three-item version of the BHS in their study on a clinical sample of 300 individuals. Three items with highest correlations with the BHS total score, plus an item # 2 from the Beck Depression Inventory (BDI), which refers to hopelessness, were included in this brief BHS. Scores on this scale were highly correlated with scores on the original scale (*r* = .88) and had relatively high internal consistency (Cronbach’s α coefficient: *r*= .80). More recently, Fraser, Burnell, et al. (49) developed two short hopelessness measures by re-wording two items of the BHS negatively (Brief-H-Neg) and items positively worded (Brief-H-Pos), and shifting the response format from “yes/no” to 5-point Likert scale (from “absolutely agree “to” absolutely disagree”). Nevertheless, no strong methodological evidence (i.e., construct validity) can be found in the literature for the Brief-H-Pos/Neg short forms. In addition, these two short forms could potentially be affected by the reverse-item bias, which is very common in scale with Likert response format (50).

### Aims

Short versions of the BHS have all been created according the Classical Test Theory (CTT) (31, 48, 49), despite the fact that the use of CTT has been criticized (51–53). The Item Response Theory (IRT) approach to the refinement of measures of clinical constructs has many practical advantages (54). For example, IRT methods could: (i) detect subtile changes in patients’ mental health that would not be recorded with the use of the mean or summed scores; (ii) overcome the sample dependence found in CTT; and (iii) produce invariant item/person statistics that allow optimal individual scores and comparison of individual scores across different tests (55, 56).In addition, applying item response models to the validation of psychopathology measures can help build a “*reasonably coherent unidimensional scale*” [(57), p.475] and treat symptoms as ordinal indicator of risk scaled along a single continuum. The unidimensional assumption was rarely met (58), especially using the CTT framework. The BHS, in this context, is not an exception. Thus, applying IRT models to the BHS could improve Beck’s conceptualization of hopelessness as a unidimensional measure.

Therefore, the purposes of the current study were: (1) to investigate the psychometric properties of the individual items of the BHS; (2) to develop a reliable and valid version including a reduced set of items since time-effective instruments would be of great practical value both in clinical and research settings; and (3) to test the diagnostic performance of the proposed short version of the BHS in classifying psychiatric inpatients at higher risk of suicide, and to compare its performance with the original 20-item BHS and to other short versions proposed in the literature (31, 48). The versions proposed by Fraser, Burnell, et al. (49) were excluded in the comparison analysis due to their methodological weaknesses and the lack of studies in support of their psychometric validity.

## Methods

### Participants and Procedure

The sample included 492 psychiatrically hospitalized, adult patients of whom 48.8% were males, with a mean age of 39.09 (SD = 13.13) years. Participants were recruited from January 2014 to December 2018 at psychiatric units situated in Sant’Andrea Medical Center, an affiliate of “Sapienza” University of Rome, Italy.

Inclusion criteria were to be inpatients aged 18 years or over with current psychiatric diagnosis performed according to the criteria of Diagnostic and Statistical Manual of Mental Disorders, Fourth Edition, Text Revision (DSM-IV-TR). Diagnoses were made by expert clinicians within the first 48 h of the psychiatric hospitalization. These were supported by means of an examination according to the Mini International Neuropsychiatric Interview criteria (59) and administered a psychological battery of tests, including the BHS, to assess the severity of psychopathology, and the presence of risk factors for suicide. The information was retrieved from clinical files for the indicated period of time. Participants with cognitive impairment and degenerative neurological disease were excluded from the study.

Primary psychiatric diagnoses included 174 (35.4%) patients with Bipolar Disorder type I (BD-I), 52 (10.6%) patients with diagnosis of Bipolar Disorder type II (BD-II), 58 (11.8%) patients with Major Depressive Disorder (MDD), 83 (16.9%) patients with Psychosis, 66 (13.4%) patients with schizoaffective disorder, 41 (8.3%) with other Axis I disorders, and 18 (3.7%) with no DSM-IV-TR diagnosis. In the total sample, 21 (4.3%) had a Personality Disorder (PD) as a secondary DSM-IV-TR psychiatric diagnosis.

The patients participated voluntarily and provided written informed consent. The study protocol was reviewed and approved by the local research ethics review board, with assurance that data would be reported anonymously and in aggregate form. All procedures were in accordance with the ethical standards of the 1964 Declaration of Helsinki and its later amendments.

### Measure

#### Beck Hopelessness Scale (BHS)

The BHS is composed of 20 dichotomous “true/false” items that aimed to assess three major aspects of hopelessness: feelings about the future, loss of motivation, and expectations. Total scores were created by first reverse-coding nine items (items 1, 3, 5, 6, 8, 10, 13, 15, 19) and then summing the item scores. Higher total scores indicate greater hopelessness (range 0–20). The Italian version of the BHS has been translated and validated by Innamorati, Lester, et al. (60) with the permission of Pearson Education (Upper Saddle River, NJ 07458, USA). A series of studies has shown that the BHS performed similarly across psychiatric inpatients and outpatients and medical samples (23–25, 36).

### Data Analysis

The Mokken Scale Analysis (MSA) was carried out within the framework of Non-parametric Item Response Theory (NIRT), in order to (a) evaluate the fundamental measurement properties of the BHS; (b) address dimensionality issue problems raised from previous research; and (c) refine the scale by providing a unidimensional, brief and reliable measure. Compared to the parametric IRT models, the Mokken probabilistic approach does not required strict assumptions about the data, and persons are allocated to a finite number of discrete ability levels. Thus, the relationship between the latent variables and the probability for a response were not required to match a specific shape (61). For this reason, Mokken’s model has been considered as less parsimonious than Rasch model. However, as pointed out by Emons, Sijtsma and Meijer (62) and Wind (63), the application of parametric IRT models (the Rasch model) might lead to inappropriate conclusions in: a) diagnosing psychological latent variables that are not clearly understood, and b) assessing the monotonicity assumption when it does not hold for a particular item (63). In this view, Mokken non-parametric models represents a viable alternative to Rasch model (64).

The MSA for dichotomous response items includes the evaluation of two models: the Monotonic Homogeneity Model (MH) and the Double Monotonicity Model (DM) (61, 65–67). Briefly, the MH model entails an ordinal scale person measurement (68), which means that the relative ordering of psychiatric inpatients on the hopelessness latent variable is invariant across items. Data are found to fit the MH model if three underlying assumptions are satisfied: *Monotonicity*, *Unidimensionality*, and *Local independence*.

The DM model represents a special case of the MH model. In our case, assessing the DM model means that all the BHS items were ordered in the same way across the psychiatric inpatients. In addition to the MH assumptions, a fourth assumption is required for the DM model: the *Invariant Item Order* (IIO). Since the DM model provides evidence for invariant ordering of items and sample for dichotomous items, this model best represents the ordinal version of the Rasch model or the 1PL-IRT (69, 70).

Like other IRT models (e.g., the Rasch model), the MSA involves an iterative process in which an observed pattern of responses is refined in order to reach the overall fit to the model expectations.

Following Sijtsma, Meijer (71) and Sijtsma and Molenaar (65), a series of steps were carried out in order to assess both the MH and the DM models as well as the scale properties (i.e., the reliability). All analyses were performed using the *Mokken* package of R (72, 73).

- The *Unidimensionality* assumption was assessed by performing the Automated Item Selection Procedure (AISP) algorithm using consecutively different values of c (.30, .35, .40, .45, .50) (74). Once a scale was detected, the scalability coefficients for individual items (H_i_), item pairs (H_ij_), and for the total scale (H) were computed, along with the standard error. For H and H_i_, values of H ≥.3 identify a “sufficient” scalability. For H_ij_, values greater than 0 and positive identify good scalability (61).
- *Local independence* was investigated using the Straat, van der Ark (75) conditional association procedure and the *W*_1_ and *W*_3_ indices developed in the *Mokken* package. The procedure identifies locally dependent item pairs.
- The *Monotonicity* of the Item Response Function (IRF) was assessed using a non-parametric regression method (76). To evaluate if IRFs were non-decreasing and monotonic, we took into account the size of the violation of monotonicity (#vi/#ac) which should not exceed the value of .3; and the Diagnostic Crit Value (crit) which should not exceed the value of 80.
- The *Invariant Item Ordering (IIO)* assumption was investigated using the method MIIO (Manifest IIO) (77). Violations of the IIO were assessed by taking into account the size of “#vi/#ac” and the “Crit” indexes. When the IIO has been established, the coefficient H^T^ expresses the precision of the item ordering (from 0 “weak” to 1 “high precision”, with a minimum value of .3).
- *Reliability* was assessed using the Moolenar–Sjitsma method (MS) (78). Cronbach (79) alpha and the Latent Class Reliability Coefficient (LCRC) (80) were also computed.
- Next, we compared the resulting unidimensional shortened version of the BHS with the Hungarian 4-item BHS models developed by Aish and Wasserman (31) and Aloba, Akinsulore (48), in order to assess which of the three competitive brief versions of the BHS performs better in measuring Beck’s Hopelessness.

The diagnostic performance of the refined 9-item BHS was assessed using the Area Under (AUC) the receiver operating characteristic curve (ROC). The Youden (J) method was employed in order to detect the cut-off score of the final item set, and we also computed key predictive statistics, including sensitivity, specificity, positive predictive value (PPV), and negative predictive value (NPV).ROC curve analysis was done using the MedCalc software package (MedCalc software, Mariakerke, Belgium) (81).

Optimal values of AUC ranged from 0 “weak performance” to 1”perfect performance” (82), with a recommended value of >.70 (83). The MINI Suicidal Subscale (59) cut-off score was employed to classify participants with high and moderate suicidal risk. We also computed a series of pairwise comparison of ROC curves to test whether the BHS long and short forms differed in performance across diagnoses. Finally, we tested the diagnostic accuracy of the 9-item BHS in discriminating between inpatients with and without prior suicide attempts.

## Results

### Mokken Analysis

First, we re-coded all the BHS items written in a reversed format. Inadmissible scores as well as missing data were removed from the dataset. Next, we submitted the BHS items to a Mokken analysis to verify scalability and the unidimensionality assumption. As shown in **Table 1**, the individual item scalability (H_i_) of the 20-item BHS was below the accepted cut-off of 30 for items #1, 3, 5 and 13. Several H_ij_ coefficients were found to be negative and below the .03 cut-off (e.g., the paired items 1-3; 3-5; and 13-2). The H coefficient of .323 (± .02) suggested a ‘*weak*’ scale and, therefore, was likely multidimensional. As expected, the AISP, with different values of lower bound, suggested a three-scale structure. The main scale was composed of 16 items identified as the “Hopelessness” dimension, while the remaining scales were small and composed of two items for each (scale 2: item 8 and 13; scale 3: items 1 and 3). However, the results confirmed the BHS as a unidimensional scale since the typical outcome pattern was confirmed (65, 84) and was observed using the AISP algorithm with different values of *c*. Hence, four items were discarded from the full 20-item scale and the remaining items were submitted to a MSA to explore the fit both of the MH (unidimensionality, monotonicity, local independency) and the DM (invariant item ordering, IIO) Mokken’s model, as well as to measure the reliability of the scale.

**Table 1:** Descriptive statistics of the items and the scale for the 20-item-BHS, the refined 9-item and the Aish et al. (31) and Aloba et al. (48) 4-item models.

	20-item BHS model			refined 9-item model			Aish et al. (31) 4-item model			Aloba et al. (48) 4-item model		
	Scale	Hi	SE	Scale	Hi	SE	Scale	Hi	SE	Scale	Hi	SE
BHS2	1	.363	.025	1	.459	.032						
BHS4	1	.350	.046									
BHS5	1	.283	.030									
BHS6	1	.403	.038	1	.459	.052	1	.500	.056			
BHS7	1	.399	.023				1	.402	.035			
BHS9	1	.308	.027				2	.298	.041	1	.166	.041
BHS10	1	.304	.028									
BHS11	1	.393	.025	1	.489	.031						
BHS12	1	.397	.029	1	.499	.034						
BHS14	1	.389	.045	1	.505	.053						
BHS15	1	.349	.025				1	.363	.038	1	.327	.035
BHS16	1	.327	.027	1	.475	.035						
BHS17	1	.346	.026	1	.497	.033						
BHS18	1	.491	.028	1	.566	.036						
BHS19	1	.383	.031									
BHS20	1	.393	.026	1	.551	.032						
BHS8	2	.221	.029							1	.259	.036
BHS13	2	.291	.042							1	.402	.052
BHS1	3	.061	.032									
BHS3	3	.088	.038									
H (SE)	.323 (.017)			.500 (.024)			.375 (.034)			.275 (.033)		
H^T^	.37			.42			.34			.24		
MS	.87			.86			.64			.53		
α	.86			.86			.60			.51		
LCRC	.89			.89			.63			.56		

Hj, item scalability coefficient; SE, Standard Error of item scalability; MS, Molenaar–Sijtsma method; α, Cronbach’s alpha; LCRC, Latent Class Reliability Coefficient.

For the 16 item-BHS scale, the H-coefficient was .42 (± .02), and H_i_ coefficients ranged from .323 (.034; item 5) to .554 (.029, item 18). All H_ij_ coefficients were non-negative, but some paired items showed scalability coefficients below the threshold (H_ij_ >.30). The conditional association procedure used to detect local independency suggested that the item pairs (4 with 7-11-12-14-18-19; 5-18; 6-18; and 14 with 14-18; 16-20; 19-18) were positively locally dependent. Next, the data analysis supported monotonicity, since no monotonicity violations were detected across all the items. Non-significant IIO was identified for items 12 and 6, and backward selection suggested removing item 7 (#vi/#ac = .20; crit = 73) and item 5 (#vi/#ac = .16; crit = 70), both of which showed signs of violating item ordering close to the recommended thresholds. The remaining items showed crit values <40, that indicated the violations reported were potentially due to sampling variations. The #vi/#ac values ranged from .9 to .2. H^T^ was .373 indicating low accuracy of the item ordering. Reliability estimates were satisfactory, with an MS index of .87, a Cronbach α of .86, and an LCRC of .89. Taken together, these results provided evidence for the weak unidimensionality of the Hopelessness scale, as it was composed at this stage. The MH and the DM Mokken’s model requirements were partially meet since neither local independency nor IIO was reached at this stage.

Next, we removed in turn the items labelled as locally dependent through the conditional association procedure and with the lower H_i_ values, and the data set was iteratively reanalyzed. Then we removed items that showed the greatest violation of item ordering.

### The Refined 9-Item Hopelessness Model

The refined Hopelessness scale resulted in a unidimensional set of nine items (items 2, 6, 11, 12, 14, 16, 17, 18, 20). The H-coefficient was .50 (± .02), all H_i_ coefficients were greater than .46, and all H_ij_ coefficients were non-negative. No violations of local independency and monotonicity were identified. A non-significant IIO was identified for all the items, and backward selection did not suggest removing any items. H^T^ was .42 indicating medium accuracy of the item ordering. These results suggested that the refined Hopelessness scale was unidimensional and met the requirements of a MH and DM Mokken scale, although the scale’s ability to discriminate between levels of hopelessness severity among psychiatric inpatients was medium. Concerning the scale properties, reliability estimates were satisfactory with an MS index of .86, a Cronbach α of .86, and an LCRC of .89.

### Comparison of Brief Versions of the BHS

Finally, the Aish and Wasserman (31) and Aloba, Akinsulore (48) 4-item versions were submitted to the MSA in order to test which of the brief version best measured hopelessness. Concerning the Aish and Wasserman (31) model, the AISP algorithm suggested a two-dimensional scale structure. The main scale was composed of items 6, 7 and 15, while item 9 loaded on a separate dimension. The H-coefficient was .37 (± .03), all H_i_ coefficients were greater than .23, and all H_ij_ coefficients were non-negative, with item 6 (SE =.05) falling below the cut-off if the standard error was taken into account. No violations of monotonicity were identified, and no local dependence issue was observed. A significant violation of the IIO was identified for item 9, and backward selection suggested removing it from the model. H^T^ was .34 indicating poor accuracy of the item ordering. Reliability analysis suggested sufficient reliability for the scale, with an MS of .64, a Cronbach α of .60, and an LCRC of .63. Taken together, the brief version of BHS proposed by Aish and Wasserman (31) satisfied the monotonicity and item local independency assumptions of Mokken analysis, but failed to address unidimensionality and the invariant item ordering, but displayed a sufficient reliability score.

With regard to the Aloba, Akinsulore (48) 4-item version, the H-coefficient was .27 (± .03). Only two H_i_ coefficients were greater than .30, and all H_ij_ coefficients were non-negative, with all the items (SE ranged from .05 to .09) falling below the cut-off if the standard error was taken into account. No violations of monotonicity were identified, and a significant IIO was identified for items 9, 8 and 15. H^T^ was .24, indicating unacceptable accuracy of the item ordering. In addition, a local dependence issue was observed between item 15 with items 9–13. The reliability analysis also suggested poor reliability for the scale, with an MS of .53, a Cronbach α of .51, and an LCRC of .56. Thus, the Aloba, Akinsulore (48) model cannot be considered as a Mokken’s reliable and valid measure of Beck’s Hopelessness, and so we eliminated this scale from subsequently analyses.

In conclusion, the refined 9-item model proposed here best represents a reliable and Mokken’s suitable measure of Hopelessness compared to the Aish and Wasserman’s (31) brief version.

### ROC Curve Analysis

A first ROC curve analysis was performed to compare the psychiatric inpatients with a high risk of suicide versus the low risk group. The results indicated that the 9-item BHS scale was able to discriminate between the two groups. The AUC for the 9-item BHS total score was .708 (95%CI =.665–.748), suggesting good discrimination between the groups. The Youden index of .39 for the 9-item BHS total score was observed at a score of 3 points, corresponding to a sensitivity of 68.56% and specificity of 64.43%. Positive and negative predictive power were 55.6% and 75.9%, respectively.

Similarly, a second ROC curve was performed to compare the psychiatric inpatients with a medium risk of suicide versus the low risk group. The results indicated that the 9-item BHS scale was able to discriminate the two groups with an AUC of .522 (95%CI of .477–.567). The Youden index of .13 for the 9-item BHS total score was observed at a score of 1 point, corresponding to a sensitivity of 90.91% and specificity of 22.22%. The positive and negative predictive powers were 7.8% and 97.1%, respectively. Thus, better accuracy was displayed by the 9-item BHS brief version in correctly diagnosing psychiatric inpatients at high risk of suicide compared to those with a medium risk of suicide.

When we compared the predictive validity of the total scores of the BHS long form and the brief 9 and 4-item models, the AUCs were identical, and the differences among them were not found to be significant for those with a high risk of suicide (ΔAUC ranged from .003 to .019) or for those with a medium risk of suicide (ΔAUC ranged from .008 to .046). Thus, results of the pairwise comparison revealed that proposed 9-item BHS brief version did not differ in diagnostic accuracy from the 20-item long form or the Aish et al.’s 4-item short form. Indicators of the predictive accuracy of the BHS scales are shown in **Table 2**.

**Table 2:** Area Under the Curve (AUC) of the Receiver Operating Characteristic Curve (ROC) Analyses for the different version of the BHS, and Comparison of independent ROC curves.

MINI Suicidality Subscale – cut-off			N	AUC	SE	(95%CI)	Sensitivity/Specificity	Cut-off	PPV/NPV	Model	ΔAUC	SE	(95%CI)	z	p
High Risk			194												
20-item BHS				.724	.023	.682–.763	59.79/72.82	>8	58.9/73.6	BHS20 vs BHS9	.016	.011	−.005–.038	1.440	.150
9-item BHS				.708	.024	.665–.748	68.56/64.43	>3	55.6/75.9	BHS20 vs BHS4	.019	.013	−.006–.045	1.461	.144
4-item BHS				.705	.023	.662–.745	59.28/69.46	>1	55.8/72.4	BHS9 vs BHS4	.003	.017	−.032–.038	.181	.856
9-item BHS							N+	N-							
Inpatients with no attempt			55	.775	.078	.645–.875	11	46		with vs no attempt	.055	.135	−.209–.320	.409	.683
Inpatients with attempt			32	.720	.110	.527–.867	21	9							
Moderate Risk			33												
20-item BHS				.560	.047	.515–.604	81.82/32.90	>4	8.1/96.2	BHS20 vs BHS9	.037	.022	−.006–.081	1.674	.094
9-item BHS				.522	.047	.477–.567	90.91/22.22	>1	7.8/97.1	BHS20 vs BHS4	.008	.031	−.054–.072	.276	.782
4-item BHS				.569	.047	.524–.613	57.58/59.26	>1	9.2/95.1	BHS9 vs BHS4	.046	.043	−.039–.031	1.066	.286
9-item BHS							N+	N-							
Inpatients with no attempt			78	.529	.123	.393–.663	6	51		with vs no attempt	.081	.231	−.370–.533	.354	.723
Inpatients with attempt			9	.611	.195	.417–.783	3	27							

Concerning the ability of the 9-item BHS into discriminate between subgroups of inpatients with or without suicide attempts, no differences were found in the AUC. The results indicated that the 9-item BHS brief version was able to detect with the same accuracy psychiatric inpatients with versus without any previous suicide attempt.

## Discussion

The BHS has been recognized as a powerful tool for predicting suicidal risk in patients diagnosed with depression mood (19, 85–87). The BHS has been used extensively under the assumption that it captures a single dimension, hopelessness, composed of three components: affective, motivational, and cognitive (6). However, international studies have reported that the BHS could consist of from one (31) to four dimensions (28). It should be noted that these differed for label and items composition.

In the current study, we submitted the BHS to a Mokken Scale Analysis (MSA) as a method to overcome the limitations of the CTT. The MSA allows the ordering of individuals on the basis of their raw scores and addresses the BHS unidimensional issue. Not surprisingly, we found that all the BHS 20 items did not tap a single unidimensional factor, but rather formed three dimensions.

The analysis did not support the original affective, motivational and cognitive model, or a clear single dimension of hopelessness. In line with a study by Aish and Wasserman (31), most of the item tapped a single dimension (Scale 1 with 16 items). Scale 2 contained items 8 (cognitive domain) and 13 (affective domain), while Scale 3 was contained items 1 (affective domain) and 3 (motivational domain). The H values were stable with respect to the scalability level for items in the Scale 1. Items that made up Scale 2 and 3 were found to be not scalable, suggesting that these scales were a weak indicator of hopelessness or that the item wording is poor. Likewise, local independency nor IIO assumptions were reached for several items at this step of the analysis. This implies that the individual’s responses to BHS items were dependent on the individual’s level of the latent trait being measured (88), as well as the ordering of the items according to its severity (or mean score) being different (not invariant) for individuals at different trait levels (89). Consistent with these results, both the MH and DM Mokken model assumptions have not been reached or met for the 20-item BHS version in the present sample of Italian psychiatric inpatients.

In order to obtain a conceptually clear measure of hopelessness, we removed items with low scalability, local dependency and a not invariant item ordering. After removing eleven items, the one-scale model maintained its psychometric viability. This was not at all obvious, given that removing items means obtaining less information for each individual and may impair construct validity and reliability (54, 62). The process resulted in a unidimensional set of nine items (items 2, 6, 11, 12, 14, 16, 17, 18, 20) and the assumptions of local independency and monotonicity for the MSA were satisfied, as well as the invariant item ordering feature. Six items corresponded to the motivational component of hopelessness, as conceived by Beck, Weissman, et al. (6), two items were drawn from the cognitive component and a single item from the affective component. All the items showed medium to high scalability coefficients, and the ability to discriminate between psychiatric inpatients with different levels of suicide risk.

Previously, some authors have proposed extremely short versions of the BHS [e.g., 2-item version by Fraser et al. (49) and 4-item versions by Aish et al. (31) and Aloba et al. (48)]. In our sample, Aish et al.’s (31) and Aloba et al.’s (48) competing models revealed psychometric weaknesses. The IRT refined short version of the BHS proposed in our study could represent a good compromise between time costs, and measurement precision.

The development and the use of short forms of measures has encountered contradictory views in the literature on clinical assessment (90). Overall, reasons why scores on short measures are likely to have less predictive validity than scores on longer inventories concern the poor sampling of the relevant behaviors (*construct underrepresentation bias*), and the lack of interest among researchers to improve the methodology of short-form development (i.e. *random measurement error issue*) (91).

In this view, this refined 9-item BHS has been developed using a sound item-development procedure: NIRT models. As suggested by Smith, McCarthy, and Anderson (90), the IRT based approach to short-form construction can lead to a shorter assessment without all the methodological issues that are often evident within CTT.

Similar, longer scales were likely to have greater content validity and higher reliability scores. To date, the overlap of content validity and scale length makes it difficult to determine “*whether the improved criterion validity of longer scales is the result of the improved reliability of measurement or the result of greater content validity*” (p. 884) (91). For example, the use of 1-item and 2-item measures has been found to increase both the Type 1-2 error rates, while slightly longer scales were found to increase the validity of study findings (92).

Similar to the content of long versions, our 9-item BHS tapped all the aspects of hopelessness: affective (item #6), motivational (item # 2–11–12–17–20), and cognitive (item #14–18) ones. Not surprisingly, the motivational aspect of this construct had greater weight, with five items that assess giving up (i.e. “*deciding not to want anything*”). As reported above, Hill, Gallagher et al. (27) found that only this was significantly related to suicidal intent. Indeed, “*loss of motivation has been found to represent the clinical picture of giving up, unpleasantness and darkness, which is a reality in suicide attempters*” (p. 142) (93).

The IRT-refined short version of the BHS also had good discriminant validity in categorizing psychiatric inpatients with high or medium suicidal risk, and patients with and without suicide attempts. Indeed, differences in diagnostic accuracy among the original 20-item version of the BHS, the four-item versions, and the IRT-refined nine-items short BHS were not significant. For high suicidal risk, a cut-off value scores >3 (with scores >1 for a moderate risk) for the refined nine-item short version of the BHS seem suitable, and this suggests that all patients with a total score of 3 should be referred for further risk assessment and management. Our IRT-refined short BHS had high sensitivity (>.90) and could be used as a valid screening tool for medium risk of suicide assessment across psychiatric inpatients.

Our results should be considered in light of four limitations. First of all, this sample consisted of a heterogeneous sample of adult psychiatric inpatients with and without suicidal attempts. Results using a more homogeneous sample of patients with mood disorders could differ, and the diagnostic accuracy of out IRT-refined nine-item short BHS reported in our study might be specifically related to the present population used. Authors of study Reise and Waller (94) showed that items selected by NIRT models were able to discriminate within a particular range of the latent dimension or in a specific population of interest. This means that if one is interested into monitoring hopelessness in a population of healthy people, it is appropriate to have items that discriminate in the low-to-average trait range.

Second, for some authors, the Mokken analysis represents an explorative approach to the development and validation of clinical scales (95, 96). For example, Meijer and Baneke (97) recommended using NIRT models to investigate the data structure and to understand how items were functioning before applying parametric IRT. Future studies may apply parametric IRT models (e.g., two parameter [2PL] and four parameter [4PL] models) to test if the behavior of the specific responses may assume a specific logistic curve. For example, applying a 4PL model to the BHS items could reveal that the probability that individuals with severe hopelessness trait manifest a specific symptom less than the 100%.

Third, social desirability or other distortions in test responses could affect self-report measures and consequently our results. Fourth, we did not investigate the predictive validity, or further important aspects of validity of this IRT-refined 9-item short BHS. Establishing predictive validity between the self-report or screening tool and a criterion measure becomes mandatory (98). Future studies are necessary to test the present refined measure with an already well-established measure, i.e. to predict suicidal ideation and/or attitude in medical inpatients and outpatients.

## Conclusions

Since its development, the Beck Hopelessness Scale was tested across different patient groups. Its shortening without a substantial loss of its predictive validity would be extremely useful in vulnerable patients, such as those to which it is addressed.

Nine best-fitting items of the Beck Hopelessness Scale satisfied the assumptions of local independency, monotonicity, and invariance of the item ordering when all the items were submitted to Mokken Scale Analysis in a large sample of adult psychiatric inpatients.

The IRT-based 9-item BHS showed good discriminant validity in categorizing psychiatric inpatients with high/medium suicidal risk and patients with and without suicide attempts, with high sensitivity (>.90). Thus, it could be used as a valid screening tool for suicidal risk assessment among psychiatric inpatients.

To our best knowledge, this study is the first focused on the application of the Item Response Theory approach to the refinement and shortening of the BHS. Previous short versions of this scale were developed within the Classical Test Theory. However, with the Item Response Theory it is possible to build a reasonably coherent unidimensional scale whose items/symptoms can be treated as ordinal indicators of the theoretical construct of hopelessness, scaled along a single continuum.

## Data Availability Statement

The datasets analyzed in this article are not publicly available. Requests to access the datasets should be directed to MI, marco.innamorati@unier.it; MP, maurizio.pompili@uniroma1.it.

## Ethics Statement

The study protocol was reviewed and approved by the local research ethics review board (Sant’Andrea Medical Center, an affiliate of the Sapienza University of Rome, Italy). The patients/participants provided their written informed consent to participate in this study.

## Author Contributions

LC and MB designed the study and conducted the statistical analyses. LC, MI, MP, and MB interpreted the data. LC, MB, MI, and DL drafted the manuscript. MP and MI recruited the sample and collaborated in editing the final manuscript. All authors contributed to the article and approved the submitted version.

## Conflict of Interest

The authors declare that the research was conducted in the absence of any commercial or financial relationships that could be construed as a potential conflict of interest.

The handling editor is currently co-organizing a Research Topic with one of the authors, MP, and confirms the absence of any other collaboration.
